# Data on the annealing of NbTiVZr at 1200 °C with slow cooling rate

**DOI:** 10.1016/j.dib.2019.103921

**Published:** 2019-04-16

**Authors:** C. Parkin, D.J.M. King, A.J. Knowles, A. Couet

**Affiliations:** a Department of Engineering Physics, University of Wisconsin-Madison, Madison, WI 53715, USA; b Centre for Nuclear Engineering, Imperial College London, South Kensington, London, SW7 2AZ, UK

## Abstract

The data presented here is complementary to the publication entitled “High temperature, low neutron cross-section high-entropy alloys in the Nb-Ti-V-Zr system” [1]. A homogenization methodology with slower cooling rate (∼2 °C/min) was performed. X-ray diffraction and scanning electron microscopy (backscattered electron and energy dispersive spectroscopy) data pertaining to annealed high-entropy alloy composition NbTiVZr is presented.

**Table:** Specifications table

Subject area	*Materials science*
More specific subject area	*Nuclear materials, high entropy alloys*
Type of data	*X-ray diffraction (XRD) intensity plots, scanning electron microscopy (SEM) micrographs, energy dispersion spectrograph EDS maps, EDS point-and-shoot compositions*
How data was acquired	*XRD was performed using a Bruker D8 Discover X-ray diffractometer, SEM/EDS was performed using a Zeiss LEO scanning electron microscope*
Data format	*XRD: background subtracted and smoothed. EDS: no standards*
Experimental factors	*Annealed in vacuum furnace w/slow cooling rate (1200*°*C, 2 °C/min)*
Experimental features	*Bulk arc melted samples of NbTiVZr were annealed at 1200*°*C and furnace cooled. The resultant microstructure was analyzed using standard laboratory equipment.*
Data source location	*University of Wisconsin- Madison, Madison, USA*
Data accessibility	*Data within this article*
Related research article	*D.J.M. King, S.T.Y. Cheung, S.A. Humphry-Baker, C. Parkin, A. Couet, M.B. Cortie, G.R. Lumpkin, S.C. Middleburgh, A.J. Knowles, Acta Mater. 166 (2019) 435–446.*

**Table:** 

**Value of the data** • Single phase high-entropy alloys are of technological importance due to the simpler microstructure allowing for easier workability and ductility. This data provides the scientific community with the knowledge that a single-phase is not achievable at ∼2 °C/min for the NbTiVZr HEA. • In combination with similar work on this system [1], [2], this data can be used to determined what the threshold cooling rate is required to achieve a single-phase. • This data is also qualitatively useful for diffusivity research as it shows that the sluggish diffusion, often claimed a HEA property, does not overpower the thermodynamically-driven chemical segregation of NbTiVZr at low temperatures.

## 1 Data

**Fig. 1:**
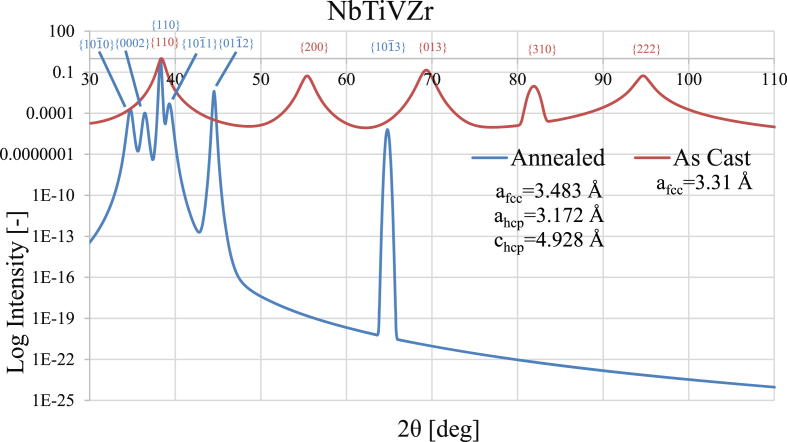
XRD pattern for as-cast (red line) and annealed (blue line) NbTiVZr. Raw data was background subtracted and peaks were fitted to Pearson VII shape functions using PeakFit v4.

**Fig. 2:**
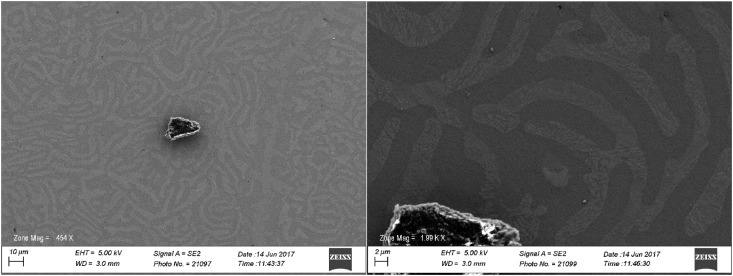
SEM BSE images of NbTiVZr sample in the post-annealed condition.

**Fig. 3:**
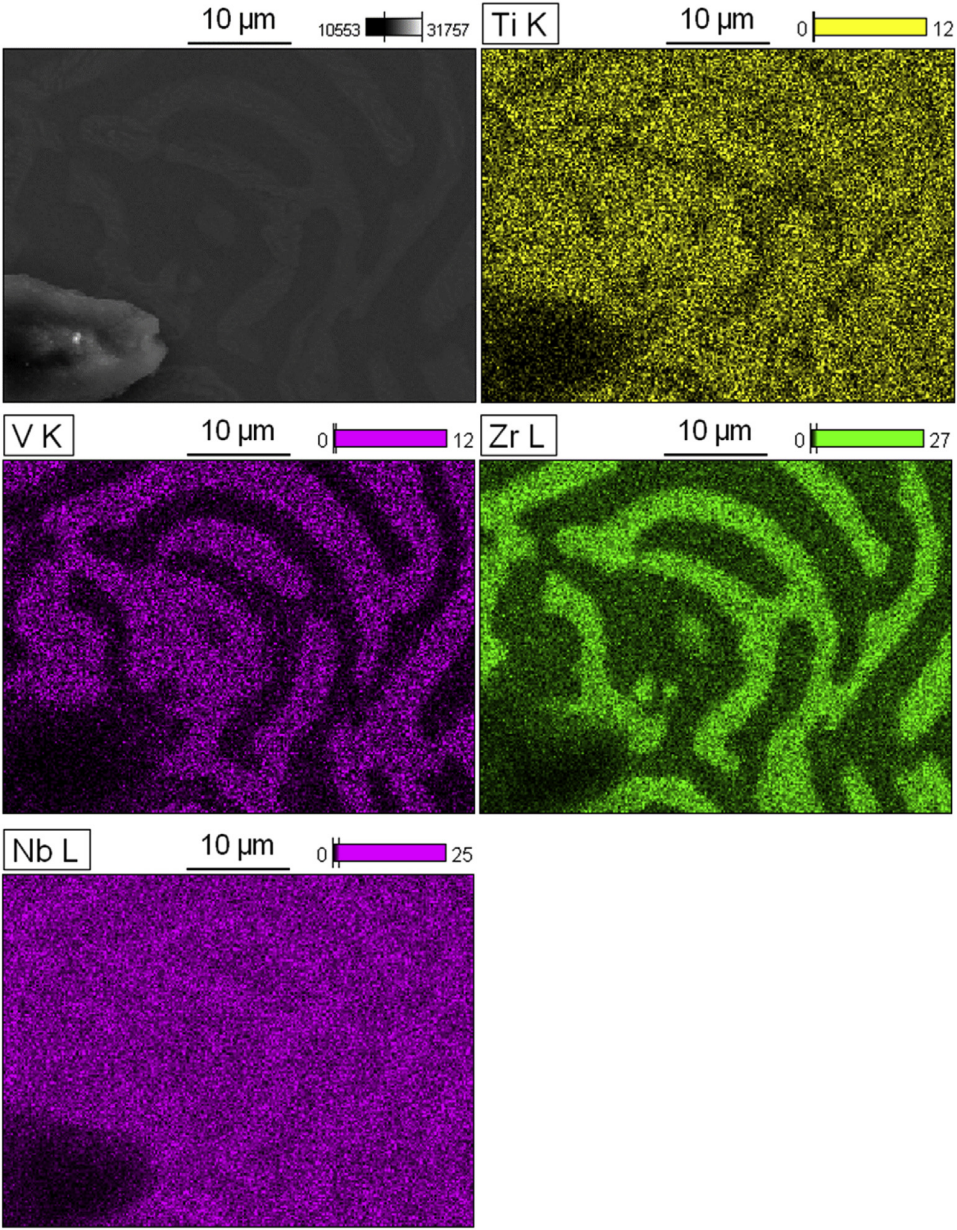
SEM-EDS images of the NbTiVZr post-annealed microstructure.

**Fig. 4:**
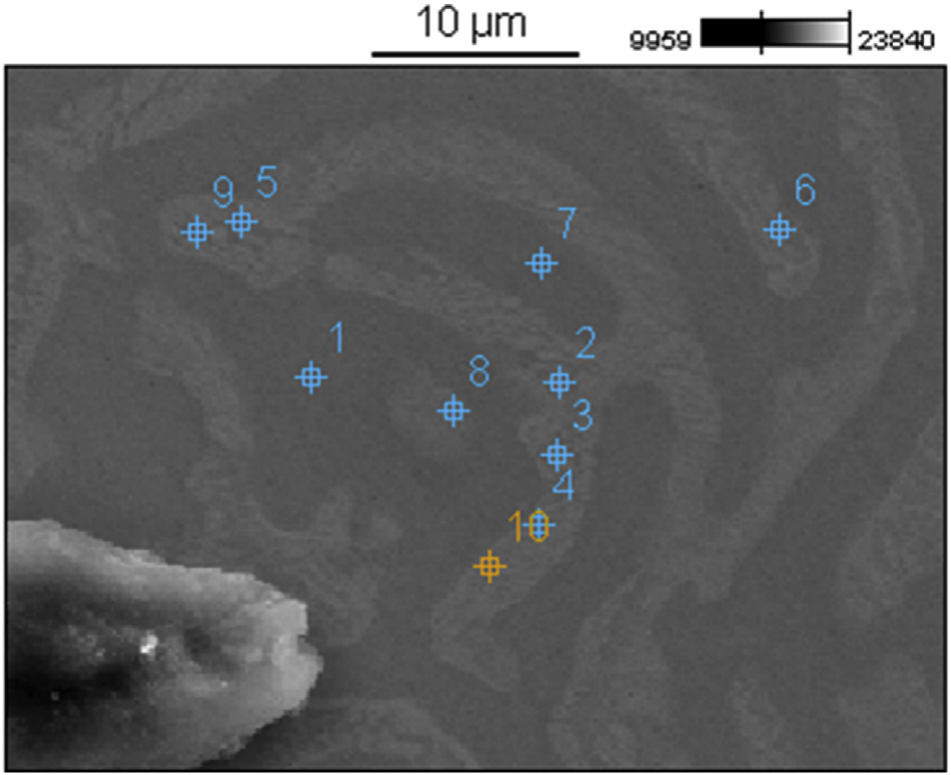
Position of point scans corresponding to Table 1.

X-ray diffraction (XRD), and scanning electron microscopy energy dispersive spectroscopy (SEM-EDS) were used to characterize the HEA composition NbTiVZr (chosen for investigation due to its relatively low thermal neutron absorption cross-section and potential use as a nuclear fuel cladding [1]). The bulk sample was annealed at 1200 °C for 24 hours followed by cooling at a rate of ∼2 °C/min. Fig. 1 shows the background subtracted and fitted XRD pattern with a normalized logarithmic scale for as-cast and annealed samples. The average composition of the annealed specimen was estimated by an area scan in a 100 μm^2^ section of the sample, see Table 1. The microstructure of the annealed specimen is provided in Fig. 2, Fig. 3, Fig. 4 with further chemical analysis in Table 2.

**Table 1:** Average composition of bulk annealed sample.

Average annealed composition (at. %)			
Ti	V	Zr	Nb
23.8 ± 0.1	24.0 ± 0.1	26.6 ± 0.1	25.7 ± 0.2

**Table 2:** Measured atomic % of each element at points corresponding to Fig. 4.

Location	Region	Ti	V	Zr	Nb
Point 1	β	25.02 ± 0.39	28.95 ± 0.43	11.38 ± 0.35	34.64 ± 0.37
Point 2	α_1_	16.95 ± 0.36	10.47 ± 0.22	60.62 ± 0.59	11.96 ± 0.49
Point 3	α_1_	16.00 ± 0.35	7.88 ± 0.20	65.88 ± 0.53	10.24 ± 0.38
Point 4	α_1_	17.11 ± 0.37	10.91 ± 0.35	58.53 ± 0.51	13.45 ± 0.39
Point 5	α_2_	30.37 ± 0.41	6.89 ± 0.20	48.46 ± 0.46	14.28 ± 0.37
Point 6	α_2_	26.34 ± 0.41	6.72 ± 0.33	53.25 ± 0.48	13.69 ± 0.38
Point 7	β	24.69 ± 0.38	29.55 ± 0.43	11.44 ± 0.34	34.33 ± 0.37
Point 8	α_1_	20.50 ± 0.37	21.52 ± 0.40	34.26 ± 0.43	23.72 ± 0.38
Point 9	α_2_	29.46 ± 0.42	8.74 ± 0.34	47.11 ± 0.53	14.70 ± 0.47
Point 10	α_1_	20.05 ± 0.38	13.23 ± 0.36	49.84 ± 0.48	16.89 ± 0.39

## 2 Experimental design, materials, and methods

The NbTiVZr sample was cut into a 1 cm diameter disk and polished. XRD was performed using a Bruker D8 Discover X-ray diffractometer using copper K-α x-rays of wavelength 1.54 Å. The primary body centred cubic (bcc) peaks were identified and smoothed using Lorentz peak fitting software PeakFit v4 and CrystalDiffract, see Fig. 1 for the fitted XRD peaks. Additional peaks were suggestive of hexagonal closed packed (hcp) alpha as well as secondary bcc phases, these were studied in further detail following alternative heat treatment in the associated work by King et al. [1].

Half of the sample was wrapped in Ta foil to prevent leaching and annealed at 1200 °C for 24 hours in a Carbolite CTF 12 vacuum tube furnace, the sample was allowed to furnace cool at ∼2 °C/min. XRD was performed again. A Zeiss LEO scanning electron microscope scanning electron equipped with EDS was used to analyze the microstructure of the annealed specimen, see Table 1 and Fig. 2, Fig. 3, Fig. 4.

EDS scans show that the annealing with slow cooling rate induced not one instance of chemical segregation into two regions either enriched or depleted in Zr (α vs. β) but also further separation of the α (lighter) region into two distinct compositions. Points 5, 6, and 8 were placed over the slightly lighter darker regions within the α phase and yielded a higher concentration of Ti and slightly lower concentration of Zr. These two regions were denoted α_2_ while the region with the highest Zr concentration was denoted α_1_. It is predicted that this secondary phase separation occurred at a lower temperature than the first separation due to the slow cooling rate.
