# Supplementary material for: Data on the annealing of NbTiVZr at 1200 °C with slow cooling rate
Source: Data Brief. 2019 Apr 16;24:103921. doi: 10.1016/j.dib.2019.103921 (PMC6488565; doi:10.1016/j.dib.2019.103921)
Supplement: Supplementary file 1 — Multimedia Component 1 [file mmc1.pdf]

CONFLICT OF INTEREST DECLARATION FORM

I, Dr Daniel Joseph Miksevicius King, wish to confirm that there are no known conflicts of interest associated with this publication and there has been no significant financial support for this work that could have influenced its outcome. I confirm that the manuscript has been read and approved by all named authors and that there are no other persons who satisfied the criteria for authorship but are not listed. I further confirm that the order of authors listed in the manuscript has been approved by all involved parties. I confirm that we have given due consideration to the protection of intellectual property associated with this work and that there are no impediments to publication, including the timing of publication, with respect to intellectual property. In so doing I confirm that we have followed the regulations of our institutions concerning intellectual property. I am the sole contact for the Editorial process (including Editorial Manager and direct communications with the office) and responsible for communicating with the other authors about progress, submissions of revisions and final approval of proofs. I confirm that we have provided a current, correct email address which is accessible by myself and which has been configured to accept email from daniel.king@imperial.ac.uk.

Sincerely,

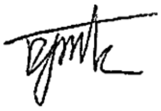

Daniel J M King
Postdoctoral Research Associate
Centre for Nuclear Engineering
Imperial College London
